# Impulse control under emotion processing: an fMRI investigation in borderline personality disorder compared to non-patients and cluster-C personality disorder patients

**DOI:** 10.1007/s11682-019-00161-0

**Published:** 2019-07-18

**Authors:** Linda van Zutphen, Nicolette Siep, Gitta A. Jacob, Gregor Domes, Andreas Sprenger, Bastian Willenborg, Rainer Goebel, Oliver Tüscher, Arnoud Arntz

**Affiliations:** 1 Department of Clinical Psychological Science, Faculty of Psychology and Neuroscience, Maastricht University, PO Box 616, 6200 MD Maastricht, the Netherlands; 2 Department of Clinical Psychology and Psychotherapy, University of Freiburg, Freiburg, Germany; 3 Department of Psychology, Laboratory for Biological and Personality Psychology, University of Freiburg, Freiburg, Germany; 4 Freiburg Brain Imaging Center, University Medical Center Freiburg, Freiburg, Germany; 5 Department of Biological and Clinical Psychology, University of Trier, Trier, Germany; 6 Departments of Neurology and Psychology, University of Lübeck, Lübeck, Germany; 7 Department of Psychiatry and Psychotherapy, University of Lübeck, Lübeck, Germany; 8 Department of Cognitive Neuroscience, Maastricht University, Maastricht, the Netherlands; 9 Department of Neuroimaging and Neuromodeling, Netherlands Institute for Neuroscience, Royal Netherlands Academy of Arts and Sciences (KNAW), Amsterdam, the Netherlands; 10 Department of Psychiatry and Psychotherapy, University Medical Center Mainz, Mainz, Germany; 11 Department of Clinical Psychology, University of Amsterdam, Amsterdam, the Netherlands

**Keywords:** BPD, Response inhibition, Impulsivity, Emotion, Neuroimaging

## Abstract

Impulsivity is a characteristic syndromal and neurobehavioral feature of borderline personality disorder (BPD). Research suggests an important interaction between high negative emotions and low behavioral inhibition in BPD. However, knowledge about the generalizability across stimulus categories and diagnosis specificity is limited. We investigated neural correlates of hypothesized impaired response inhibition of BPD patients to negative, positive and erotic stimuli, by comparing them to non-patients and cluster-C personality disorder patients. During fMRI scanning, 53 BPD patients, 34 non-patients and 20 cluster-C personality disorder patients completed an affective go/no-go task, including social pictures. BPD patients showed more omission errors than non-patients, independent of the stimulus category. Furthermore, BPD patients showed higher activity in the inferior parietal lobule and frontal eye fields when inhibiting negative versus neutral stimuli. Activity of the inferior parietal lobule correlated positively with the BPD checklist subscale impulsivity. When inhibiting emotional stimuli, BPD patients showed an altered brain activity in the inferior parietal lobe and frontal eye fields, whereas previously shown dysfunctional prefrontal activity was not replicated. BPD patients showed a general responsivity across stimulus categories in the frontal eye fields, whereas effects in the inferior parietal lobe were specific for negative stimuli. Results of diagnosis specificity support a dimensional rather than a categorical differentiation between BPD and cluster-C patients during inhibition of social emotional stimuli. Supported by behavioral results, BPD patients showed no deficiencies in emotionally modulated response inhibition per se but the present findings rather hint at attentional difficulties for emotional information.

**Electronic supplementary material:**

The online version of this article (10.1007/s11682-019-00161-0) contains supplementary material, which is available to authorized users.

## Introduction

One of the hallmarks of borderline personality disorder (BPD) is impulsivity; commonly associated with self-injury, anger outbursts, substance abuse, unprotected sex, excessive spending, reckless driving, and uncontrolled eating (American Psychiatric Association 2013; Sebastian et al. 2013a). Impulsivity is a broad concept often defined as a pattern of behavioral disinhibition, including a predisposition of rapidly and unplanned responses without considering the consequences (Moeller et al. 2001). Hence, impulsivity has often been related to response inhibition difficulties.

Behavioral data resulting from BPD studies investigating the hypothesized impaired response inhibition, using *go/no-go tasks*, are inconsistent (Sebastian et al. 2013a). Some studies did not report differences between BPD and non-patients (NPC) (Vollm et al. 2004; van Eijk et al. 2015; Soloff et al. 2015), while others showed that BPD patients make significantly more commission errors, supporting that they are indeed worse in response inhibition (Leyton et al. 2001; Rentrop et al. 2008; Mortensen et al. 2010). Neuroimaging studies examining response inhibition in BPD are also inconclusive. An fMRI study in BPD patients compared to NPC showed a negative correlation between the number of commission errors and activation in the medial frontal gyrus, anterior cingulate cortex (ACC), temporal gyrus and striatum (Leyton et al. 2001). Two other fMRI studies did not show any differences in brain activity between BPD and NPC (Mortensen et al. 2010; van Eijk et al. 2015).

In response to the conflicting results, it has been suggested that BPD patients act impulsive especially in negative emotional conditions (Domes et al. 2006; Jacob et al. 2010; Sebastian et al. 2013a; Sinke et al. 2017). This interaction between negative emotions and impulsivity has been examined in two fMRI studies using go/no-go tasks (Jacob et al. 2013; Silbersweig et al. 2007). Silbersweig et al. (2007) reported a decreased ventromedial prefrontal cortex (vmPFC) and increased lateral orbitofrontal cortex (OFC) and dorsolateral PFC (dlPFC) activity in BPD compared to NPC during response inhibition of negative words. Additionally, in BPD a negative correlation between impulsivity scores and vmPFC activity was shown (Silbersweig et al. 2007). Another study (Jacob et al. 2013) examined response inhibition after anger induction and found in BPD compared to NPC a decreased inferior frontal cortex (IFC) and increased nucleus subthalamicus activity. Since no significant group differences were shown at behavioral level, the authors suggested a compensatory mechanism involving the subthalamic nucleus for the missing IFC activity (Jacob et al. 2013). Together, negative emotions might interfere with behavioral inhibition and underlie impulsivity in BPD, represented by heightened activity in brain areas responding to emotions accompanied by decreased activity in brain areas that control behavioral impulses.

This study is part of an international multicenter RCT on group schema therapy versus treatment-as-usual in which the BPD patients participated (Wetzelaer et al. 2014). The first aim of the present study is to examine neurocircuits involved in response inhibition in BPD. Both previous studies exclusively reported negative with neutral stimuli contrasts. Accordingly it remains unclear whether impaired response inhibition solely involves negative emotions, or whether it generalizes and also holds for positive emotions, pointing to a general impaired response inhibition. Furthermore, given that history of sexual traumatization and intimacy is often reported in BPD (Zanarini et al. 2002), erotic content might trigger negative emotional reactions leading to impulsive behavior. Consequently, erotic stimuli were included, of which we expected to evoke emotional responses similar to the negative stimuli in BPD. Additionally, because of high rates of comorbid Axis I and II disorders (Zanarini et al. 1998a, b; Zimmerman and Mattia 1999; McGlashan et al. 2000) within BPD it remains uncertain whether findings are diagnosis specific or characteristic of psychopathology in general. By adding a clinical control group (CCP) the second aim of the present study is to examine BPD-specificity of impulsivity. Motivated by prior work it was hypothesized that BPD patients have more problems with inhibiting their response under emotional states compared to both control groups, related to higher activity in emotion-related brain areas (e.g. amygdala, ventral striatum, anterior insula) (Goldstein et al. 2007; Shafritz et al. 2006) and dysregulated activity in areas related to response inhibition (e.g. IFC/ventrolateral PFC (vlPFC), dorsal ACC, dlPFC, vmPFC, inferior parietal lobe (IPL), pre-supplementary motor area, thalamus, dorsal striatum, nucleus subthalamicus) (Goldstein et al. 2007; Sebastian et al. 2013b; Simmonds et al. 2008; Swick et al. 2011; Shafritz et al. 2006; Jacob et al. 2013; Silbersweig et al. 2007; Leyton et al. 2001).

## Material and methods

### Participants

The present study complements our recent study that examined stimulus category specificity and diagnosis specificity of neural correlates of emotional regulation in BPD (van Zutphen et al. 2017). Fifty-nine BPD patients, 41 NPC and 29 CCP underwent current scanning session, of which 53 BPD patients, 34 NPC and 20 CCP met scanning and clinical criteria and were left for the analyses.

Participants were recruited from two sites in the Netherlands (Maastricht, Heerlen) and three sites in Germany (Freiburg, Hamburg, Lübeck). Patients were recruited from the mental health clinics at local sites. BPD patients were recruited within the context of an international multicenter RCT on group schema therapy versus treatment-as-usual (Wetzelaer et al. 2014). NPC were recruited among the general population at each site via postings and personal contacts. Participants had to be hetero- or bisexual females, aged 18–65, and have sufficient understanding of the language at the local site. Only females were chosen since gender might influence emotional processing (Whittle et al. 2011), and because in mental health care BPD is more often diagnosed in females. We excluded homosexual females, because we used heterosexual erotic stimuli. Since impulsivity is strongly related to attention-deficit/hyperactivity disorder (ADHD) patients with comorbid ADHD were excluded. ADHD was screened with the World Health Organization Adult Self-Report Scale (ASRS-v1.1; Kessler et al. 2005), if positive diagnosis was checked with the SCID for childhood diagnoses (KID-SCID; Smith et al. 2005). General exclusion criteria were lifetime psychotic or bipolar disorder type-I, dissociative identity disorder, serious and/or unstable medical illness, substance dependence needing clinical detoxification and fMRI exclusion criteria (i.e. claustrophobia, metal objects, cardiac arrhythmia, epilepsy, tattoos at neck/head and pregnancy).

BPD and CCP patients underwent the Structural Clinical Interview (SCID) for Axis I (First et al. 1994) and II (First et al. 1997) assessed by trained interviewers and were diagnosed according to the DSM-IV criteria. Preferably measurements were collected before start of patients’ therapy, unless impossible due to scheduling problems fMRI-measurements had to be finished within three months from the start of therapy (*n* = 14; 73.55 ± 58.18 days). BPD patients that scored full or sub-threshold on narcissistic and antisocial PD were excluded for reasons related to the clinical trial in which this study sample participated (Wetzelaer et al. 2014). Moreover, BPD patients were further screened by means of BPD Severity Index (Arntz et al. 2003; Giesen-Bloo et al. 2010; Kroger et al. 2013), for inclusion this score was >20 (31.36 ± 6.86). CCP were not allowed to score full or sub-threshold Cluster-B PD, or > 2 BPD criteria. Additionally, CCP were excluded if they scored above 100 on the BPD checklist, as this implies BPD-pathology. Non-patients (NPC) did not meet current diagnostic criteria for any Axis I or II disorder. They were screened with the SCID I and II screeners (First et al. 1994, 1997), positive items on the screeners were checked with SCID interviews. Furthermore, to distinguish non-patients from patients, NPC were not allowed to score above 0.70 on the Brief symptom inventory.

We additionally assessed the Brief Symptom Inventory (Derogatis 1993), BPD checklist (Arntz and Dreessen 1995) and Interview for Trauma Events in Childhood (Lobbestael et al. 2009). For demographic and diagnostic variables of all groups see Table 1. We attempted to recruit both control groups in a similar range as the BPD group on age, intelligence and handedness in terms of means and variance. No significant group differences were shown for age, handedness and IQ. Relative to both control groups, BPD patients scored significantly higher on the impulsivity subscale of the BPD checklist (Table 1). See online resources for additional details regarding participant recruitment and measure descriptions.

**Table 1:** Demographic and diagnostic variables of the three groups: borderline personality disorder (BPD), non-patient controls (NPC), and cluster-C control patients (CCP)

	BPD	NPC	CCP	Test statistics	
	(*n* = 53)	(*n* = 34)	(*n* = 20)	*F*	*p*
Age, years, mean (SD)	31.02 (8.77)	29.44 (11.31)	29.20 (9.80)	0.388	0.679
Education level^a^, No. (%)				4.14^b^	0.126
Level 1	12 (22.6)	7 (20.6)	3 (15.0)		
Level 2	8 (15.1)	2 (5.9)	4 (20.0)		
Level 3	15 (28.3)	4 (11.8)	5 (25.0)		
Level 4	2 (3.8)	2 (5.9)	3 (15.0)		
Level 5	13 (24.5)	14 (41.2)	3 (15.0)		
Level 6	3 (5.7)	5 (14.7)	2 (10.0)		
Estimated IQ^c^, mean (SD)	96.43 (9.88)	98.82 (11.05)	98.02 (9.86)	0.597	0.552
Handedness, No. L/?R/M	3/46/3	1/33/-	-/20/-	4.76^d^	0.313
BSI, mean (SD), total	1.74 (0.56)	0.14 (0.15)	1.07 (0.45)	132.04	<0.001^e^
BPD checklist, mean (SD), total	119.92 (25.03)	51.26 (6.46)	74.26 (18.22)	133.09	<0.001^f^
Subscale impulsivity, mean (SD)	15.75 (5.19)	9.76 (1.16)	10.75 (1.97)	29.42	<0.001^g^
ITEC, mean (SD)				9.22	<0.001^h^
Sexual abuse	9.02 (9.05)	0.11 (0.39)	1.33 (3.53)	19.76	<0.001
Physical abuse	17.26 (11.79)	1.64 (3.58)	5.99 (9.24)	27.30	<0.001
Emotional abuse	20.24 (8.78)	2.47 (3.48)	13.33 (8.77)	50.71	<0.001
Emotional neglect	11.31 (6.75)	0.80 (1.94)	6.03 (6.68)	31.52	<0.001
Physical neglect	10.24 (9.14)	0.96 (2.95)	4.50 (7.08)	15.24	<0.001
Dissociation, mean (SD)				6.90	<0.001^i^
prior scanning	19.09 (19.89)	2.37 (2.49)	7.36 (10.86)	13.84	<0.001
post scanning	26.20 (23.15)	4.96 (7.55)	10.61 (14.90)	14.90	<0.001
Anxiety, mean (SD)				6.84	<0.001^j^
prior scanning	26.10 (25.55)	3.18 (5.46)	15.65(21.34)	12.86	<0.001
post scanning	18.94 (24.40)	1.88 (1.93)	7.55 (9.61)	10.09	<0.001
Nervousness, mean (SD)				7.10	<0.001^k^
prior scanning	32.75 (27.31)	5.26 (9.63)	19.70 (22.46)	15.64	<0.001
post scanning	20.77 (24.79)	2.88 (3.55)	11.95 (17.11)	9.09	<0.001
Axis I disorders, No. (%)					*p*^l^
Major depressive disorder	47 (88.7)		12 (60.0)		0.006
Dysthymic	4 (7.5)		1 (5.0)		0.701
Bipolar type II	1 (1.9)		–		0.536
Generalized anxiety disorder	1 (1.9)		–		0.536
Panic disorder with agoraphobia	7 (13.2)		1 (5.0)		0.317
Panic disorder	7 (13.2)		3 (15.0)		0.843
Agoraphobia	3 (5.7)		–		0.277
Specific phobia	10 (18.9)		–		0.037
Social phobia	18 (34.0)		5 (25.0)		0.462
Obsessive compulsive disorder	8 (15.1)		1 (5.0)		0.242
Posttraumatic stress disorder	20 (37.7)		2 (10.0)		0.021
Somatoform disorder	5 (9.4)		4 (20.0)		0.221
Eating disorders	20 (37.7)		7 (35.0)		0.829
Substance abuse	26 (49.1)		1 (5.0)		0.001
Intermitted explosive disorder	1 (1.9)		–		0.536
Axis II disorders, No. (%)					
Avoidant PD	26 (49.1)		14 (70.0)		0.109
Dependent PD	9 (17.0)		2 (10.0)		0.457
Obsessive compulsive PD	10 (18.9)		6 (30.0)		0.305
Passive aggressive PD	4 (7.5)		–		0.206
Depressive PD	15 (28.3)		2 (10.0)		0.099
Paranoid PD	15 (28.3)		–		0.008
Schizotypal PD	1 (1.9)		–		0.536
Schizoid PD	1 (1.9)		–		0.536
Medication, No. (%)					
Antidepressants	36 (67.9)		8 (40.0)		0.030
Antipsychotics	8 (15.1)		–		0.066
Hypnotics	3 (5.7)		–		0.277
Mood Stabilizers	1 (1.9)		–		0.536

Abbreviations: *L*, Left; *R*, Right; *M*, Mixed; *BSI*, Brief Symptom Inventory; *BPD* checklist, Borderline checklist; *ITEC*, Interview Traumatic Events Childhood; *PD*, Personality Disorder

^a^Level of education of both the Dutch and German educational systems were translated into the International Standard Classification of Education (ISCED), in current study six levels of education were divided ranging from lower secondary school to Master’s degree

^b^Value is based on Kruskal-Wallis

^c^Assessed with four subtasks of the WAIS

^d^Value is based on Chi-square, data of one BPD patient not available

^e^All three groups significantly differed from each other (*p* ?< ?0.001)

^f^All three groups significantly differed from each other (*p* ?< ?0.001), data of one CCP not available

^g^BPD patients significantly differed from both control groups (*p* ?< ?0.001)

^h^MANOVA and ANOVAs showed significant group effects over traumas. BPD patients experienced significantly more trauma compared to both control groups regarding sexual abuse (vs. NPC *p* ?< ?0.001; vs. CCP *p* ?< ?0.001), physical abuse (vs. NPC *p* ?< ?0.001; vs. CCP *p* ?< ?0.001) and physical neglect (vs. NPC *p* ?< ?0.001; vs. CCP *p* = 0.015). The three groups significantly differed from each other concerning emotional abuse (BPD vs. NPC *p* ?< ?0.001; BPD vs. CCP *p* = 0.003; NPC vs. CCP *p* ?< ?0.001) and emotional neglect (BPD vs. NPC *p* ?< ?0.001; BPD vs. CCP *p* = 0.003; NPC vs. CCP *p* = 0.009), with BPD patients experiencing the most trauma, followed by the CCP and the NPC experienced the least trauma. Data of five NPC and one CCP not available

^i^MANOVA and ANOVAs showed significant group effects over dissociation. BPD patients dissociated significantly more prior and post scanning compared to both control groups (prior scanning: BPD vs. NPC *p* ?< ?0.001, BPD vs. CCP *p* = 0.011; post scanning: BPD vs. NPC *p* ?< ?0.001, BPD vs. NPC *p* = 0.006). Data of six BPD patients and one CCP not available

^j^MANOVA and ANOVAs showed significant group effects over anxiety. BPD patients were more anxious compared to NPC prior scanning (BPD vs. NPC *p* ?< ?0.001) and more anxious compared to both control groups post scanning (BPD vs. NPC *p* ?< ?0.001, BPD vs. CCP *p* = 0.046). Data of five BPD patients not available

^k^MANOVA and ANOVAs showed significant group effects over nervousness. BPD patients were more nervous compared to NPC prior and post scanning (prior scanning: BPD vs. NPC *p* ?< ?0.001; post scanning: BPD vs. NPC *p* ?< ?0.001). Data of five BPD patients not available

^l^Value is based on Chi-square

Before onset of the study, written informed consent was obtained. Participants received a small financial remuneration. The study was carried out in accordance with the latest version of the Declaration of Helsinki, and approved by the local medical ethical committees (Wetzelaer et al. 2014).

### Go/no-go task

**Fig. 1:**
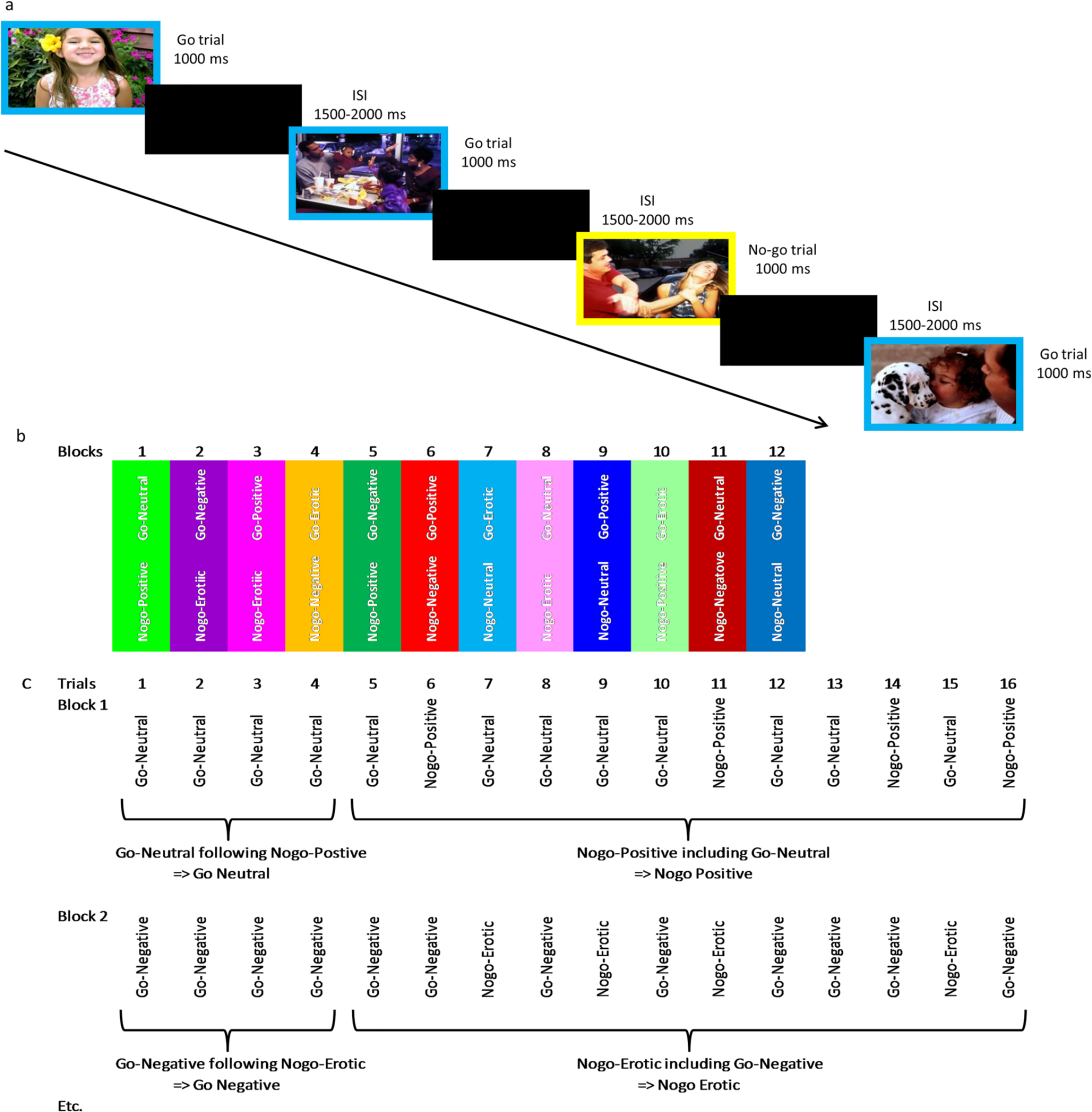
Task design. Panel **a** shows a block in which positive pictures were combined with go-trials and negative pictures with nogo-trials. Stimuli were presented for 1000 msec and followed by a variable inter-stimulus-interval (ISI) of 1500–2000 msec. Participants had to make a button press for the go trials (blue square), while they had to inhibit this motor response for the no-go trials (yellow square). Panel **b** depicts the order of the blocks and set go/nogo-combinations. Panel **c** illustrates the go and no-go blocks for statistical analyses

Participants underwent scanning while they performed a visual affective go/no-go task (Fig. 1a; adapted from Silbersweig et al. (2007)). Neutral, negative, positive and erotic pictures were presented in a blue or yellow frame. Participants were instructed to perform as fast and correctly as possible a right-index-finger button-press when the picture was framed within a blue square (go-trials), while they had to inhibit this motor response when the picture was framed within a yellow square (nogo-trials). Button-press responses and reaction times were recorded. Pictures were selected from the International Affective Picture System (Lang et al. 1997) and additional erotic pictures from Jacob et al. (2011). Only pictures with a social content were selected since BPD patients are particularly responsive to interpersonal cues (Koenigsberg et al. 2009).

The task consisted of four runs with 12 blocks per run. Each block consisted of 16 randomized trials of two stimulus categories with 25% nogo-trials and 75% go-trials. Due to the differentiation of go and no-go stimuli, and because of the four different affective picture categories, 12 go/no-go combinations were set. All combinations of picture categories were used so each nogo-trial is controlled for any other picture category (Fig. 1b). In order to induce a prepotent motor response each block started with a minimum of four go-trials. Due to technical problems behavioral data of 26 out of the 53 BPD patients, 8 out of the 34 NPC and 1 out of the 20 CCP were missing, leading resulting usable behavioral data sets for 27 BPD patients, 26 NPC and 19 CCP.

### Procedure

Prior to scanning all participants were trained on a practice task outside the scanner. This task contained novel stimuli during the experimental task inside the scanner. After finishing the practice task the participant entered the scanner and the scanning session of 75 min was completed. Presentation of the stimuli and recordings of behavioral responses were controlled by Presentation (Neurobehavioral Systems Inc., Albany, CA, USA). The visual stimuli were projected via PC and beamer onto a screen that was viewed through a mirror on the headcoil or via a goggle-system. As part of the scanning session participants also underwent two resting state scans (data reported separately). At the end of the session the participant completed an ‘exit’-questionnaire, assessing information about their general experience with the fMRI. In addition, before and after scanning the anxiety and dissociation state was assessed (Stiglmayr et al. 2001). Finally, the participants had to rate their subjective reaction of each picture they had seen during the session.

### fMRI acquisition

Functional MRI was performed on 3 T scanners at all three sites, at Maastricht on a Siemens Magnetom Allegra head-only scanner equipped with a birdcage headcoil (Siemens Medical Systems, Erlangen, Germany), at Freiburg on a Siemens tim-Trio Magnetom whole body scanner (Siemens Medical Systems, Erlangen, Germany) equipped with an 8-channel headcoil, and at Lübeck on a Philips Achiva whole body scanner equipped with an 8-channel headcoil (Philips Healthcare, Best, The Netherlands). The BPD patients from Heerlen were scanned in Maastricht and from Hamburg were scanned in Lübeck. In Maastricht 11 BPD, 10 NPC and 11 CCP were scanned, Freiburg scanned 13 BPD, 11 NPC and 6 CCP, finally 29 BPD, 13 NPC and 3 CCP were scanned in Lubeck.

Participants were scanned in head first supine position. Head movements were minimalized using foam paddings. Additionally, the participant was instructed to avoid moving as much as possible during scanning. T2*-weighted images were acquired via echo planar imaging (EPI), using the following imaging parameters: TR = 2000 ms, TE = 27 ms, flip angle = 90°, FoV = 192 × 192 mm, voxel size = 3 × 3 × 3 mm, and matrix = 64 × 64. Images were recorder in four runs of 280 images in Maastricht, 276 images in Freiburg and 256 images Lübeck. One volume in Maastricht consisted of 32, and in Freiburg and Lübeck of 34, interleaved measured axial slices. A slice tilt correction of −30° was used to optimize the susceptibility and minimize the distortion artifacts within the amygdala the T2*-weighted images (Morawetz et al. 2008) in Maastricht and Freiburg. A whole-brain anatomical scan in sagittal plane was acquired, using a high resolution T1-weigthed sequence (TR = 2250 ms, TE = 2.6 ms, flip angle = 9°, FoV = 256 × 256 mm, voxel size 1 × 1 × 1 mm). In total, 192 images were obtained in Maastricht, 160 in Freiburg and 170 in Lübeck.

### fMRI preprocessing

Preprocessing and statistical analyses were performed with BrainVoyager 2.6 (Brain Innovation, Maastricht, The Netherlands). The first two images of each run were discarded because of saturation effects. Preprocessing contained slice time correction with sinc interpolation, 3D motion correction for three translation and three rotation parameters with trilinear interpolation for detection and sinc interpolation for motion correction and removal of low-frequency drifts was performed by high-pass temporal filtering of 2 sines/cosines per run (Goebel et al. 2006). To improve data quality anatomical scans were peeled from the skull and corrected for intensity inhomogeneities. Participants underwent another session concerning a different task (data presented elsewhere van Zutphen et al. 2017), in which also an anatomical scan was conducted. To obtain a high resolution and high contrast anatomical scan, both anatomical scans were averaged when possible. After preprocessing the functional data were coregistered with the anatomical data per run, and for each run a volume-time-course was created. Each volume-time-course was spatially smoothed with a 6 mm full-width-at-half-maximum isotropic Gaussian kernel. Spatial normalization was performed using standard Talairach transformation procedures (Talairach and Tournoux 1988).

### Data analyses

To model the hemodynamic nogo-response the first four go-trials of each block (to induce the prepotent response tendency) were considered as separate go-blocks, with the next 12 mixed go/nogo-trials constituting a nogo-block, this resulted in 24 block types (Fig. 1c). Since we were interested in response inhibition under emotional processing in general and not in specific go/no-go combinations the applied general linear model included eight predictors collapsed irrespective of the specific stimulus category of the go’s or no-go’s; denoting go-neutral, go-negative, go-positive, go-erotic, nogo-neutral, nogo-negative, nogo-positive, nogo-erotic. Subsequently, six motion parameters were added as confound predictors.

Differences in brain activity between BPD and NPC during response inhibition of negative stimuli were first used to define the regions of interest. In a second step we subsequently looked for the effects of positive and erotic stimuli, and CCP in these clusters. Individual statistical parametric maps were generated for hypothesis-driven contrast nogo-negative versus nogo-neutral. These contrast images were entered into group-level analyses, including group (BPD, NPC) and site (Maastricht, Freiburg, Lubeck) as between-factors. Next, a whole-brain random-effects (RFX) ANOVA was carried out including nogo-stimulus (nogo-negative vs. nogo-neutral) x group (BPD vs. NPC). The resulting F-maps were thresholded at *p* < 0.005 and corrected for multiple comparisons with a cluster-size threshold at *p* = 0.05, being 13 voxels, to balance type-I and II errors (Lieberman and Cunningham 2009). The minimal cluster-size was determined by a cluster-level estimation plugin implemented in BrainVoyager, which performs a cluster-level correction of multiple comparisons using a Monte Carlo simulation-based approach (1000 iterations: Forman et al. 1995). For each cluster beta values per predictor, per run, of each participant individually were extracted and exported to SPSS 21 (IBM Corporation, New York) for more detailed linear mixed model (LMM) analyses. To investigate how BPD-specific our results were, mean betas per cluster of the CCP were additionally extracted from the clusters and used in post-hoc comparisons. The same strategy was applied to examine the BPD response-uniqueness to negative stimuli; mean betas per cluster of the positive and erotic stimuli were extracted.

LMM-analyses were used for further analyses, to control for 1) the response of the go-trials within the blocks by using the beta values of the go’s as a time-dependent covariate, and 2) the unbalanced design (as go’s and no-go’s of the same stimulus category were never combined in one block). First-order autoregression was chosen as covariance structure for the repeated part, including run and order of the block within the run, as this led to the best fitting models. Backwards stepwise deletion was used to eliminate non-significant variables and interactions. The fixed part contained dummies of the nogo-stimulus categories, group and their interactions, and the response on the go’s and order of the block as running covariates. A random intercept and slope for run, with covariance components as covariance structure, of each participant were included as this resulted in a better fit of the models.

To relate brain activity to impulsivity, correlation analyses with the subscale impulsivity of the BPD checklist, including BPD and NPC, were conducted within the clusters that showed a significant group difference in response inhibition. Findings were considered significant at *p* < 0.05/#comparisons, based on the number of resulting brain areas per contrast.

Subsequently, to explore differences in brain activity during response inhibition of positive or erotic stimuli the same analytical procedure was performed of the significant brain areas resulting from the whole-brain analysis of the following hypothesis-driven interactions: nogo-stimulus (nogo-positive vs. nogo-neutral) x group (BPD vs. NPC), and nogo-stimulus (nogo-erotic vs. nogo-neutral) x group (BPD vs. NPC). The minimal cluster-size threshold of both resulting F-maps was 13 voxels.

## Results

### Behavioral data

**Fig. 2:**
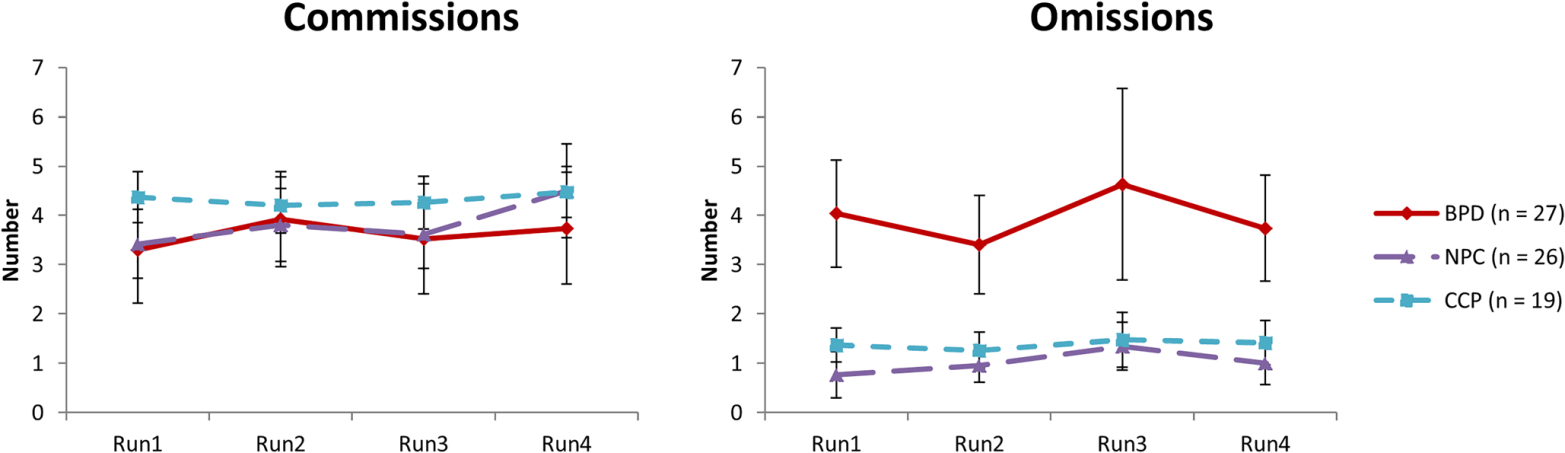
The number of commission and omission error per group across the runs. Error bars represent standard error of the mean

To examine group differences the number of commission errors (go reaction in nogo-trial), omission errors (no-go reaction in go-trial), and reaction times of commissions or hits (Table 2) four LMM-analyses were performed. No significant interactions were shown. The number of commissions, reaction times of commissions and reaction times of hits showed a main effect of stimulus category (*F*_3,2971.40_ = 3.69, *p* = 0.012; *F*_3,2984.91_ = 4.74, *p* = 0.003; *F*_3,2581.25_ = 7.56, *p* < 0.001, respectively). Post-hoc tests revealed more commissions (B = −0.061, SE = 0.019, *p* = 0.008) and longer reaction times of these commissions (B = −30.59, SE = 8.22, *p* = 0.001) for negative versus erotic stimuli. The reaction time of the positive hits were longer versus the erotic (B = −10.49, SE = 2.24, *p* < 0.001), negative (B = −6.37, SE = 2.21, *p* = 0.020), and neutral hits (B = −6.06, SE = 2.42, *p* = 0.050). The number of omissions showed a main effect of group (*F*_2,68.24_ = 4.73, *p* = 0.012), in which BPD patients committed more omissions than NPC (B = 0.138, SE = 0.049, *p* = 0.014) and CCP (B = 0.124, SE = 0.053, *p* = 0.039). (Neural correlates of group differences regarding response inhibition are reported in the Online Resource.) Additionally, we tested whether the number of commissions and omissions were related to the length of the session. For both there was no main effect of run (4 levels): commissions *F*_3,67_ = 2.60, *p* = 0.059; omissions *F*_3,67_ = 0.73, *p* = 0.541, or run x group interaction: commissions *F*_6,136_ = 1.19, *p* = 0.317; omissions *F*_6,136_ = 0.57, *p* = 0.757. Also at a more detailed level of the blocks there was no main effect (4 × 12 = 48 levels) or block x group interaction for either the commissions as well as the omissions. This indicated that errors did not occur more often at the end of the session due to stress or fatigue (Fig. 2). Moreover, this was independent of the stimulus category.

**Table 2:** Behavioral data and stimulus evaluations of the borderline personality disorder (BPD), non-patient controls (NPC), and cluster-C control patients (CCP) during the go/no-go task for the neutral, negative, positive and erotic stimuli

Behavioral data	BPD (*n* = 27)	NPC (*n* = 26)	CCP (*n* = 19)
Omissions, mean (SD)			
Neutral	2.26 (3.74)	0.62 (1.39)	0.89 (1.24)
Negative	2.22 (3.76)	0.50 (1.14)	0.89 (1.47)
Positive	1.89 (3.14)	0.73 (1.43)	0.26 (0.45)
Erotic	2.67 (3.88)	0.38 (1.06)	0.79 (1.08)
Commissions, mean (SD)			
Neutral	1.96 (1.70)	2.31 (2.92)	2.37 (2.85)
Negative	2.59 (1.91)	2.27 (2.29)	2.95 (3.46)
Positive	2.26 (2.09)	1.96 (2.11)	2.95 (2.70)
Erotic	1.78 (1.78)	1.88 (1.99)	1.95 (2.46)
Reaction time Commissions, mean (SD), msec			
Neutral	379.76 (229.74)	264.74 (210.21)	245.94 (206.00)
Negative	400.15 (172.96)	399.25 (246.29)	302.38 (229.85)
Positive	300.89 (197.59)	266.42 (194.36)	372.94 (133.98)
Erotic	313.21 (252.27)	287.53 (211.61)	245.55 (205.51)
Reaction time Hits^a^, mean (SD), msec			
Neutral	487.97 (59.32)	457.76 (60.10)	472.37 (55.72)
Negative	480.98 (57.72)	449.59 (63.13)	469.99 (58.93)
Positive	478.66 (57.70)	445.72 (59.94)	457.46 (59.61)
Erotic	484.58 (56.94)	451.96 (55.01)	466.22 (57.11)
Stimulus evaluations after scanning	BPD (*n* = 30)	NPC (*n* = 30)	CCP (*n* = 19)
Arousal, mean (SD)			
Neutral	3.73 (1.01)	2.97 (1.27)	3.63 (0.96)
Negative	5.50 (1.91)	6.13 (1.96)	6.47 (1.45)
Positive	3.63 (1.45)	3.03 (2.03)	3.47 (1.61)
Erotic	4.30 (1.49)	4.03 (1.77)	4.63 (1.50)
Valence, mean (SD)			
Neutral	5.03 (0.72)	6.00 (1.11)	5.26 (0.65)
Negative	2.23 (1.01)	2.13 (0.86)	2.05 (1.22)
Positive	7.10 (1.03)	7.97 (0.96)	7.52 (0.96)
Erotic	6.20 (1.49)	7.23 (1.38)	6.53 (1.65)

^a^Mean reaction time was calculated for the correct trials

Manipulation checks of the stimulus evaluations after scanning confirmed that negative stimuli were most arousing across groups, followed by erotic stimuli, positive and neutral stimuli were equally least arousing (*F*_3,74_ = 67.10, *p* < 0.001) (Table 2). Valence ratings showed a significant valence x group interaction (*F*_6,150_ = 2.58, *p* = 0.021). Post-hoc tests showed that BPD patients rated the erotic (*t*_58_ = −2.78, *p* = 0.007), neutral (t_58_ = −3.99, *p* < 0.001) and positive (*t*_58_ = 3.37, *p* = 0.001) stimuli significantly less pleasant than NPC. CCP rated neutral stimuli significantly less pleasant than NPC (*t*_47_ = 2.61, *p* = 0.012) (Table 2). Negative stimuli did not differ in valence rating across groups.

### Functional MRI results

**Fig. 3:**
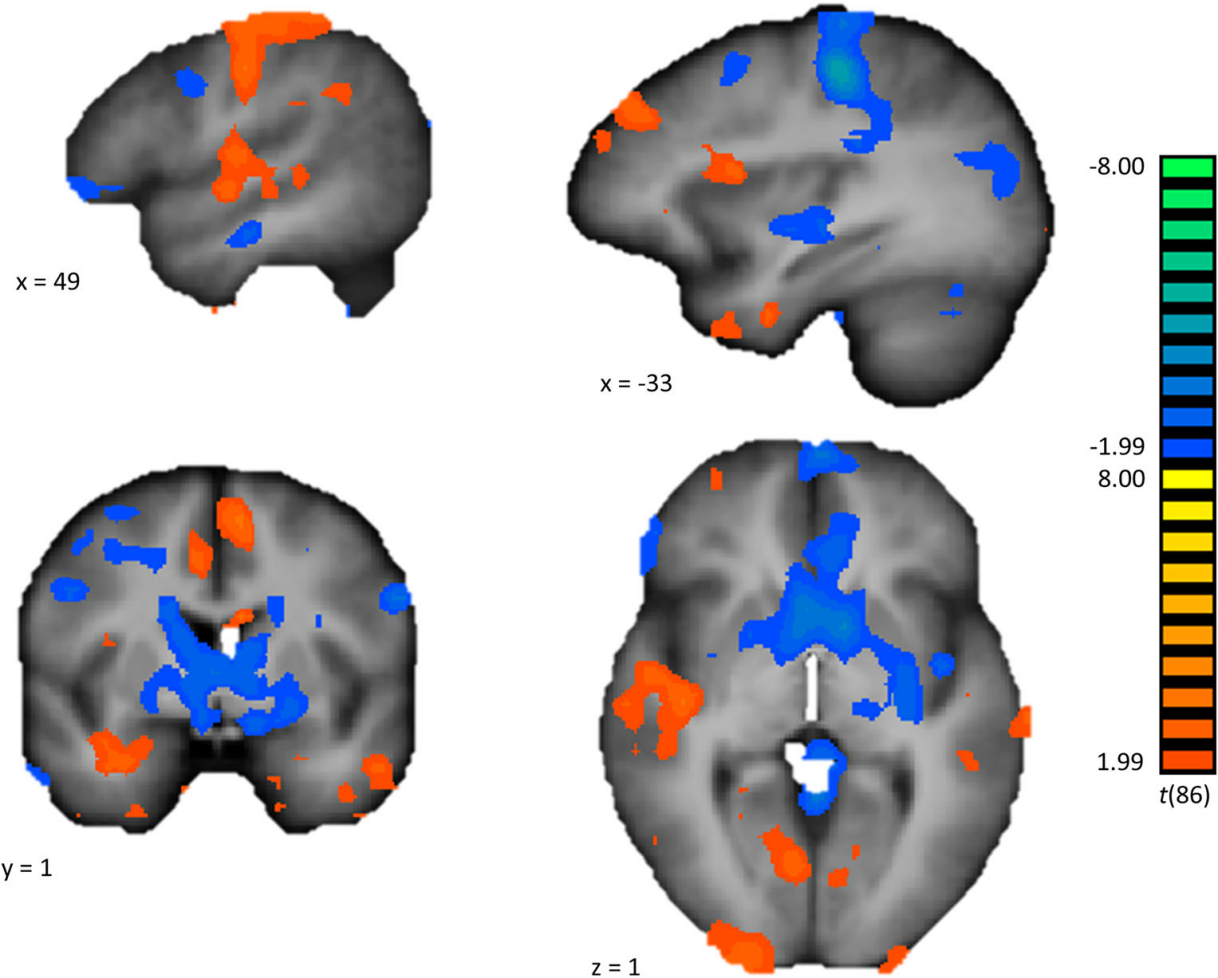
Brain activity for the contrast No-go vs. Go. The *t*-map was thresholded at *p* = 0.05 and overlaid on an average anatomical brain over all participants in Talairach space, shown in radiological convention. The hot colors indicate increased activity during the no-go blocks compared to the go blocks, and the cold colors indicate decreased activity during the no-go blocks compared to the go blocks

Brain activity for the main effect of response inhibition (contrast: no-go vs. go) independent of stimulus category including the BPD and NPC participants nicely showed the response inhibition network similarly as previously reported (Sebastian et al. 2013b; Simmonds et al. 2008; Swick et al. 2011) (Fig. 3).

**Fig. 4:**
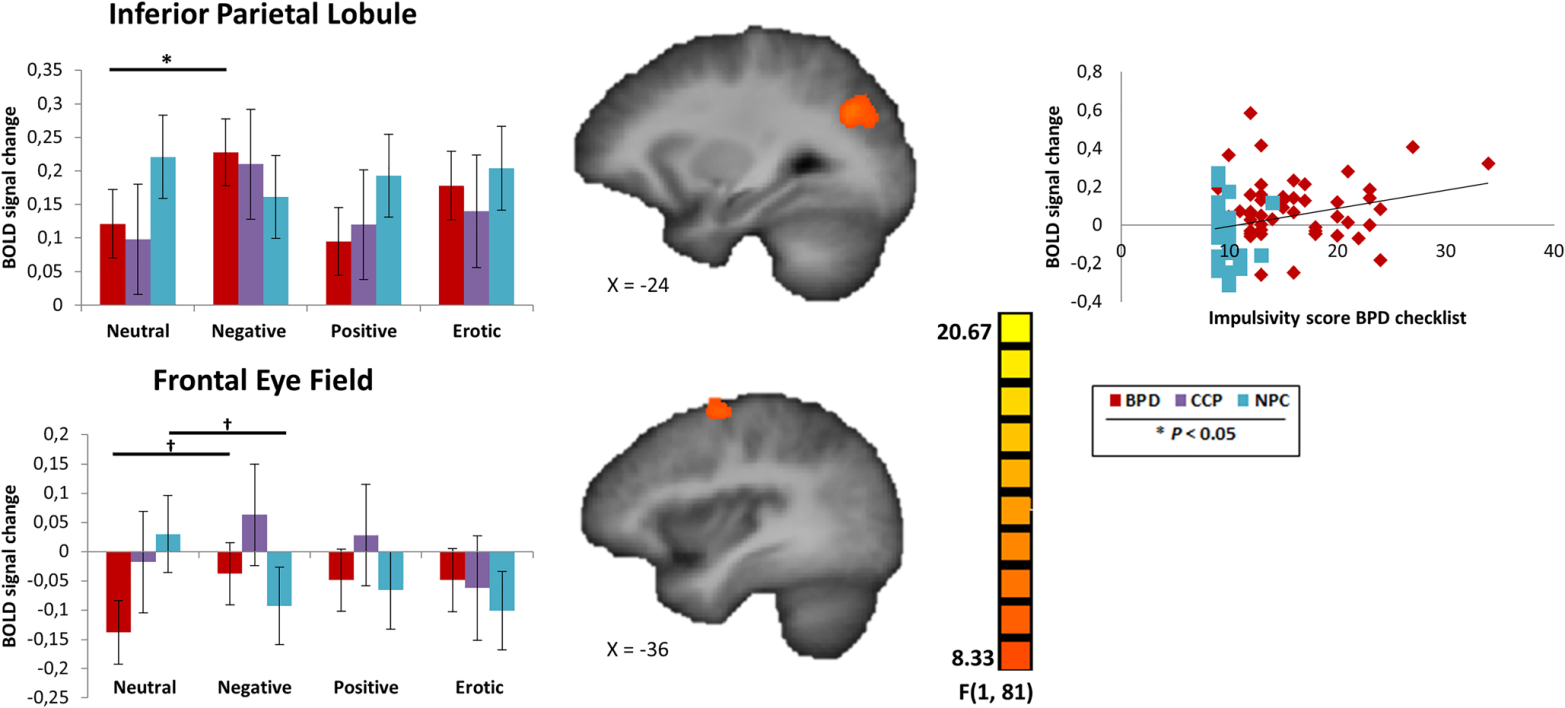
Locations of clusters resulting from the whole-brain RFX ANOVA testing differences in response inhibition. Cluster coordinates are reported in Talairach space. Bar plots represent mean estimates and standard error of beta values based on linear mixed model analyses. The scatterplot depicts correlation analyses between brain activity of the inferior parietal lobule when inhibiting negative stimuli (nogo-negative minus nogo-neutral) and impulsivity scores from the BPD checklist. † Pairwise comparisons showed marginally higher activity when inhibiting negative versus neutral stimuli in BPD *p* = 0.053, and marginally lower activity when inhibiting negative versus neutral stimuli in NPC *p* = 0.053

The RFX ANOVA F-map: nogo-stimulus (nogo-negative vs. nogo-neutral) x group (BPD vs. NPC) resulted in the left IPL and left frontal eye fields (FEF) (Table 3a). LMM-analysis showed higher activity in the IPL and FEF when inhibiting negative versus neutral stimuli in BPD (Fig. 4).

**Table 3:** Overview of fMRI results and statistical tests

A. Resulting clusters for RFX ANOVA testing differences in response inhibition between BPD and NPC																		
Brain area	L/R	BA	Cluster size	Talairach peak voxel				Nogo Negative minus Nogo Neutral			Nogo Positive minus Nogo Neutral				Nogo Erotic minus Nogo Neutral			
			mm^3^	x		y	z	B	SE	p	B		SE	p	B	SE	p	
Nogo Negative versus Nogo Neutral																		
Inferior parietal lobe	L	7	1941	-24		-64	28	-.167	.060	**.006**	< -.001		.061	.989	-.074	.062	.239	
Middle frontal gyrus, Frontal eye fields	L	8	470	-36		-4	58	-.223	.060	**<.001**	-.185		.061	**.002**	-.220	.062	**<.001**	
Nogo Positive versus Nogo Neutral																		
Posterior cingulate cortex	L	30	489	-24		-67	8	.117	.056	.037	.207		.057	**<.001**	.099	.058	.086	
Nogo Erotic versus Nogo Neutral																		
Subcallosal gyrus, ventromedial	L	25	381	-9		23	-11	.016	.061	.799	.094		.061	.126	.127	.061	**.039**	
Brainstem^a^	R		1140	3		-25	-42	.017	.047	.719	.053		.047	.260	.094	.048	**.049**	
B. Significance levels of linear and quadratic trends of brain responses in relation to severity of personality psychopathology: from NPC to CCP to BPD																		
	Nogo Negative minus Nogo Neutral						Nogo Positive minus Nogo Neutral						Nogo Erotic minus Nogo Neutral					
	Linear			Quadratic			Linear			Quadratic			Linear			Quadratic		
	B	SE	p	B	SE	p	B	SE	p	B	SE	p	B	SE	p	B	SE	p
Nogo Negative versus Nogo Neutral																		
Inferior parietal lobe	.083	.030	.006	-.029	.023	.201	<.001	.030	.989	-.016	.023	.482	.037	.031	.239	-.008	.024	.752
Middle frontal gyrus, Frontal eye fields	.112	.030	<.001	-.031	.023	.182	.092	.030	.002	-.016	.023	.481	.110	.031	<.001	.008	.023	.742
Nogo Positive versus Nogo Neutral																		
Posterior cingulate cortex	-.059	.028	.037	.020	.021	.350	-.103	.028	<.001	.058	.022	.007	-.050	.029	.086	.042	.022	.056
Nogo Erotic versus Nogo Neutral																		
Subcallosal gyrus, ventromedial	-.008	.031	.799	.010	.023	.674	-.047	.031	.126	. 014	.023	.550	-.063	.031	.039	-.043	.023	.067
Brainstem^a^	-.008	.024	.719	-.019	.018	.298	-.027	.024	.260	.006	.018	.725	-.047	.024	.049	.008	.018	.650

Abbreviations: BPD, Borderline personality disorder; NPC, Non-patient controls; CCP, Cluster-C control patients; L, Left; R, Right; BA, Brodmann area.

Mean estimates (B), standard errors (SE) and *p*-values are based on linear mixed model analyses. *F*-map thresholded at *p* < 0.005 and cluster size.

^a^Possibly affected by site.

Results showed a significant interaction for positive and erotic stimuli in the FEF (Table 3a). Pairwise comparisons showed marginally lower activity when inhibiting erotic versus neutral stimuli in NPC. The comparison BPD versus CCP did not show significant differences. In addition to the go-response, a dummy of the go-stimulus category (to equalize the go-stimulus category across the nogo-negative and nogo-neutral blocks) was added. Post-hoc sensitivity analyses with go-stimulus category as additional covariate showed similar results. Since we did not detect differences between BPD and CCP, we post-hoc tested linear and quadratic trends of brain responses in relation to severity of personality psychopathology from NPC to CCP to BPD. Both clusters support a linear relationship for the significant nogo-stimulus x group interactions over the three groups, with the CCP scoring in-between the BPD and NPC (Table 3b; Fig. 4).

The activity of the IPL during response inhibition of negative versus neutral stimuli correlated positively with the BPD checklist subscale impulsivity (*r*_85_ = 0.272, *p* = 0.011), indicating that higher impulsivity scores were associated with more brain activity when inhibiting negative stimuli (Fig. 4).

Each cluster was examined post-hoc for confounding effect of medication. LMM-analyses within BPD showed no significant [stimulus x medication (medicated vs. non-medicated)] interactions in the clusters. Moreover the results remained the same for the BPD versus CCP comparison when medication was added to the LMM-analyses including both groups.

Finally, because the age range is rather large and as a higher age is associated with lower impulsivity we post-hoc employed age as a covariate. When accounted for age in the analyses the results were similar.

### Exploratory analyses of BPD patients compared to both control groups when inhibiting positive and erotic stimuli versus neutral stimuli

The interaction regarding response inhibition of positive stimuli [(nogo-positive vs. nogo-neutral) x (BPD vs. NPC)] revealed the left posterior cingulate cortex (PCC; Table 3a). LMM-analysis showed less activity in BPD compared to NPC when inhibiting positive versus neutral stimuli. No differences were found for the comparison of BPD and CCP. The interaction concerning response inhibition of erotic stimuli [(nogo-erotic vs. nogo-neutral) x group (BPD vs. NPC)] exhibited the left vmPFC and right brainstem (Table 3a). A significant difference was found between BPD and CCP in the vmPFC (B = 0.192, SE = 0.074, *p* = 0.009), BPD showed less activity compared to CCP for erotic stimuli. Post-hoc sensitivity analyses with go-stimulus category as additional covariate showed similar results for the PCC and vmPFC. Furthermore, analyses of linear or quadratic relationship of brain responses in relation to severity of personality psychopathology showed no exclusive evidence in the PCC, whereas the vmPFC showed support for a linear relationship (Table 3b). Correlation analyses did show a significant negative association between the BPD checklist subscale impulsivity and vmPFC activity when inhibiting erotic stimuli (*r*_*85*_ = −0.289, *p* = 0.007). Finally, LMM-analyses within BPD showed no significant stimulus x medication interactions, and the results of the comparison of BPD with CCP did also hold when corrected for medication. Moreover, when age was added as covariate the results were the same.

## Discussion

The aim of the current fMRI study was to investigate stimulus category specificity and diagnosis specificity of response inhibition under emotional processing in BPD. Elaborating on previous research, we used an affective go/no-go paradigm and added positive and erotic stimuli to the traditional negative and neutral stimuli, and compared BPD to non-patient and cluster-C personality disorder groups. Behavioral data showed that BPD made more omission errors compared to NPC and CCP, whereas comparable commission errors were shown. Stimulus category had no effect on the number of omissions. Neuroimaging results showed higher activity in the IPL and FEF when inhibiting negative versus neutral stimuli in BPD. Furthermore, increased activity of the IPL correlated with higher impulsivity scores on the BPD checklist, indicating when scoring higher on the impulsivity subscale more brain activity is necessary while inhibiting negative stimuli. BPD patients showed a general responsivity across stimulus categories in the FEF, whereas effects in the IPL were specific for negative stimuli. In both clusters the comparison BPD versus CCP revealed no differences, indicating that activity in these clusters is not BPD-specific. Contrary to our expectations, these findings do not support the hypothesized impaired response inhibition in BPD.

The present study could not replicate the altered prefrontal activity (decreased vmPFC and IFC, increased lateral OFC and dlPFC) in BPD as reported in the two previous studies on BPD impulsivity (Jacob et al. 2013; Silbersweig et al. 2007). One explanation could be that we used a different go/no-go paradigm than previous studies. Silbersweig et al. (2007) used a linguistic go/no-go task, in which words were used, whereas we used pictures. Jacob et al. (2013) used a simple letter-based go/no-go task after an emotion induction, while we used a complex go/no-go task comprising emotional stimuli. Additionally, concerning the heterogeneous results BPD patients show an emotional hyperreactivity with respect to BPD-specific stimuli rather than to emotional stimuli in general (Sauer et al. 2014). Silbersweig et al. (2007) and Jacob et al. (2013) both used BPD salient stimuli, whereas we used general emotional stimuli. Moreover, the current study had more statistical power than previous studies, and since small-powered studies have low reproducibility (Button et al. 2013) it might well be that the current study did not replicate previous results. Taken together, as fMRI studies examining the hypothesized impaired response inhibition in BPD comprise a diversity of task designs, more research is necessary to explain the underlying neurocircuits.

The left FEF and left IPL are advocated to be core regions in the *left* dorsal attention network, which is involved in top-down attentional control over brain areas of the sensory cortices and ventral attention network (Corbetta et al. 2008). Attention is a bottleneck in many information processing streams, and therefore might interfere with higher cognitive processes. Hence, we speculate that the findings of altered activity of the left dorsal attention network in BPD hints towards an impaired top-down attentional bias implementation and inappropriate top-down control over the ventral attention network. This might mean that BPD patients constantly shift their attention towards unimportant stimuli and/or that they have difficulties withdrawing their attention from emotional stimuli. Subsequently, continuous reorientation and/or slowed disengagement might interfere with cognitive resources required to disengage attention from sensory salient but behaviorally irrelevant stimuli. This is in line with behavioral studies, reporting on biased attention towards emotional stimuli in BPD (Arntz et al. 2000; von Ceumern-Lindenstjerna et al. 2010; Kaiser et al. 2016; Bertsch et al. 2017). Additional evidence is provided by previous fMRI studies using attention tasks, i.e. an emotional Stroop task (Wingenfeld et al. 2009) and a Posner task (Mortensen et al. 2016), in which BPD patients showed altered attentional control networks (e.g. dACC) compared to NPC. Further this idea is supported by our behavioral data in which no group differences were shown for commissions, but more omissions were observed for BPD patients compared to NPC, indicating no deficiencies in response inhibition per se but pointing to attentional difficulties. This is in line with Silbersweig et al. (2007) who also showed more omissions for BPD patients during both negative and neutral stimuli, indicating more attentional demands for BPD patients. However, this warrants further research using attentional bias paradigms.

Investigation of diagnosis specificity indicates a linear relationship, with an intermediate response of the CCP, placed between NPC and BPD. This supports the idea that cluster-C patients exhibit common emotional and interpersonal problems with BPD (American Psychiatric Association 2013; Sharp et al. 2015), and that the observed effects are more a dimensional rather than a categorical differentiation.

Results concerning stimulus category specificity showed that the FEF also showed a significant interaction involving positive and erotic stimuli, caused by an activity modulation across stimulus categories in NPC. This modulation by positive and erotic stimuli was absent in BPD. This result indicates, in contrast to the hypotheses, that BPD is not affected by the stimulus category. However, the IPL did show higher activity during the presentation of the negative stimuli in BPD compared to the other stimuli categories. This effect was not present in the NPC. The IPL activity during response inhibition is previously linked to attentional processes to go/nogo-stimuli (Simmonds et al. 2008; Swick et al. 2011). This might suggest that in BPD attention is driven towards negative stimuli at the perceptional level of the IPL, and that BPD at the higher cognitive processing level of the FEF do not discern the stimulus categories anymore.

Results regarding response inhibition of positive and erotic stimuli revealed less activity in the PCC when inhibiting positive stimuli in BPD compared to NPC, and altered activity in the vmPFC and brainstem when inhibiting erotic stimuli. Hence, differential activity in these brain areas indicates emotional arousal to the content of the pictures during response inhibition rather than inhibitory processing.

The strengths of the current study include an extension of stimulus categories and inclusion of the clinical control group compared to previous studies (van Zutphen et al. 2015). There are also some limitations to be recognized. Firstly, only women were recruited, which limits the generalizability of our results to men. Additionally, BPD patients represented a heterogeneous group, therefore we cannot rule out the possibility that our results might be affected by the presence of comorbid diagnoses. Secondly, patients were taking psychotropic medication for clinical reasons, which is a potentially confounding factor (Delaveau et al. 2011; Ma 2015). However, excluding patients on medication would result in a non-representative and less severe clinical sample. As adding medication as a covariate removes variance associated with group differences, additional analyses within the BPD group were performed. Interactions of medication within the BPD group were not significant within any of the resulting brain areas, suggesting that medication did not influence the results. Thirdly, an unbalanced design was used in this study, in which blocks containing the same stimulus category for the go’s and no-go’s were missing and the neutral nogo-blocks were always presented in a block containing emotional go-trials. However, by using LMM we could control for the unbalanced design by adding the initial go’s brain responses as covariates. Fourth, scanner parameters across sites could not be perfectly equalized. Except for the cluster of the brainstem of the nogo-erotic versus nogo-neutral contrast, the reported clusters did not show overlap with the significant clusters of group x stimulus x site interaction at lenient significance level of *p* < 0.05 (see Online Resource). Additionally, more detailed analyses within SPSS did not show a significant group x stimulus x site interaction, again with the exception of the brainstem, and the group x stimulus remained significant after adding site and its interactions to the model. Although our results seem to show that our data are not affected by different scanner sites, there might be a variation left in the recorded data which cannot be ruled out completely. Fifth, we did not consider the menstrual cycle of the participants, which might have an effect on emotion processing and modulate brain activity (Sundstrom Poromaa and Gingnell 2014), however these effects should be random given the large sample size.

## Conclusions

In conclusion, BPD patients showed altered responses in the IPL and FEF, when inhibiting emotional stimuli. BPD patients showed a general responsivity across stimulus categories in the FEF, whereas effects in the IPL were specific for negative stimuli. Linearity analyses implied a dimensional rather than a categorical differentiation, with responses of CCP in-between NPC and BPD. In face of the current discussion on impulse control deficits in BPD (Sebastian et al. 2013a; van Eijk et al. 2015), the current results add further evidence in the view that interference control in BPD might be impaired in early processing stages, rather than in motor inhibitory control itself (Sebastian et al. 2013a; van Eijk et al. 2015).

## Electronic supplementary material

ESM 1 (DOCX 381 kb)
