# Supplementary material for: Impulse control under emotion processing: an fMRI investigation in borderline personality disorder compared to non-patients and cluster-C personality disorder patients
Source: Brain Imaging Behav. 2019 Jul 18;14(6):2107–21. doi: 10.1007/s11682-019-00161-0 (PMC7647993; doi:10.1007/s11682-019-00161-0)
Supplement: Supplementary file 1 — (DOCX 381 kb) [file 11682_2019_161_MOESM1_ESM.docx]

**Supplementary Materials**

*Brain Imaging and Behavior*

**Impulse control under emotion processing: an fMRI investigation in borderline personality disorder compared to non-patients and cluster-C personality disorder patients**

Linda van Zutphen^*^, Nicolette Siep, Gitta A. Jacob, Gregor Domes, Andreas Sprenger, Bastian Willenborg, Rainer Goebel, Oliver Tüscher, & Arnoud Arntz

^*^ Corresponding author

Dept. of Clinical Psychological Science, Faculty of Psychology and Neuroscience, Maastricht University, P.O. Box 616, 6200 MD, Maastricht, The Netherlands

Email: [linda.vanzutphen@maastrichtuniversity.nl](mailto:linda.vanzutphen@maastrichtuniversity.nl)

ORCID ID: <http://orcid.org/0000-0002-8962-7832>

**Supplementary Materials**

The text below is almost identical to van Zutphen et al. ([2017](#_ENREF_12)) due to two parallel studies performed by the same research groups.

**Methods**

**Participants**

Patients were recruited from mental health clinics at local sites, Virenze-Riagg Maastricht (The Netherlands), PsyQ Heerlen (The Netherlands), the BPD treatment unit of the Department of Psychiatry and Psychotherapy at the Medical Center Freiburg (Germany), the Department of Psychiatry and Psychotherapy at the University Hospital Lübeck (Germany) and the Institute for Behavior Therapy Training Hamburg (Germany).

Three BPD patients, one NPC and one CCP were excluded because of invalid or incomplete data, two NPC and one CCP because of too much motion (if head motion in 2 or more runs was > 4 mm) during scanning, and one NPC and two CCP because of incorrect responses on the behavioral data (i.e. commission errors were made throughout the task, possibly implying incorrect task performance), two NPC because of scores above 0.70 on the BSI and three CCP because of scores above 100 on the BPD checklist. Finally, three BPD and two CCP were excluded because they had an estimated IQ outside the range of 75-120. Additionally, as there was a disproportionate number of NPC with IQ higher than the highest IQ in the BPD group, we excluded one NPC with IQ scores above the 95-percentile to guarantee a match between NPC and BPD patients.

**Measures**

*Brief Symptom Inventory (BSI)* - The BSI is a brief psychological self-report inventory of general symptoms of psychopathology during the past week ([Derogatis 1993](#_ENREF_6)). It is a short alternative for the Symptom Checklist-90-R from which it was developed. It contains 53 items divided over nine dimensions: somatization, obsession-compulsion, interpersonal sensitivity, depressive mood, anxiety, hostility, phobic anxiety, paranoid ideation and psychoticism. Answers are scored on a 5-point Likert scale, ranging from 0 (not at all) to 4 (extremely). Scores of the dimensions are calculated by summing the values for the items divided by the number of items within the subscales. The total score measures the level of symptomatology, which is the sum of the nine dimensions plus the four additional items divided by total number of items. The internal consistency showed a Cronbach’s α of 0.96 for the total instrument and ranged between 0.71 and 0.85 for its subscales ([De Beurs and Zitman 2006](#_ENREF_5); [Derogatis 1993](#_ENREF_6)). To distinguish patients from non-patients a cutoff score of 0.70 is suggested ([De Beurs 2004](#_ENREF_4)).

*BPD Checklist* - The BPD Checklist is a self-report questionnaire used to assess the burden of BPD symptoms as experienced during the last month ([Arntz and Dreessen 1995](#_ENREF_1)). It consists of 47 items based on the nine dimensions of BPD in DSM-IV. Items must be rated on 5-point Likert scale, ranging from 1 (not at all) to 5 (extremely). Next to the total sumscore, also the scores for the nine subscales can be calculated. Scores above 100 signify BPD-pathology. When control patients showed an elevated score, an extra check with the SCID II BPD section was done.

*Interview for Traumatic Events in Childhood (ITEC)* *-* The ITEC is a retrospective, semi-structured interview to measure childhood maltreatment prior the age of 18, including sexual (12 items), physical (13 items) and emotional abuse (9 items), and emotional (6 items) and physical neglect (15 items) ([Lobbestael et al. 2009](#_ENREF_9)). For each item the participant experienced maltreatment, follow-up questions are used to gather more detailed information about the perpetrator(s), age of onset, frequency, duration of the trauma and the impact on the victim in the past and in the present. This information was used to calculate a severity score between 0 and 1, such that the score increased with the severity of the event itself, the closeness of the perpetrator, the younger age of onset, the longer duration of the event, and the higher impact on the victim. For each subscales the severity scores for the events are summed, the higher this score, the more severe the maltreatment. Internal consistencies of these scales were moderate to excellent, with Cronbach’s α varying between 0.58 and 0.89 with a mean of 0.79 ([Lobbestael et al. 2009](#_ENREF_9)). In addition to the victimization scales, similar scales were created for witnessing the various forms of maltreatment. In current study only the victim scales are used. One average the administration time is about 30 minutes but can take up to one hour in case of multiple maltreatments.

*Dissociation and Anxiety* - Present state dissociative experiences were assessed using four items of the Dissociation-Tension-Scale ([Stiglmayr et al. 2001](#_ENREF_11)), containing derealization and changes in perception of one’s body, hearing and pain. Additionally two items concerning the level of anxiety and the level of nervousness were added. Responses were indicated on a visual analogue scale, ranging from 0 (not at all) to 10 (extremely). The dissociation score was the averaged across the four dissociation items. Internal consistencies of the dissociation score proved to be good in current sample, with a Cronbach’s α of 0.86 concerning dissociation before scanning and Cronbach’s α = 0.87 concerning dissociation after scanning.

*Self-Assessment Manikin Scale* - The pictures shown during the scanning session were qualitatively assessed for valence and arousal using the Self-Assessment Manikin Scale. The Self-Assessment Manikin Scale consists of a series of human-like figures to measures the affective reaction of a person to stimuli ([Bradley and Lang 1994](#_ENREF_3)). Intensity of valence and arousal were both rated on a 9-point scale, with for valence 1 being extremely unpleasant and 9 being extremely pleasant, and for arousal 1 being most calm and 9 being most aroused.

*Wechsler Adult Intelligence Scale* - IQ was estimated by means of four subtests of the Wechsler Adult Intelligence Scale, including two verbal (i.e. Vocabulary and Similarities) and two nonverbal tests (i.e. Block design and Matrix reasoning). Together these subtests correlate strongly with general intellectual ability ([Schrimsher et al. 2008](#_ENREF_10)). IQ was estimated based on the optimized regression equation: 39.05 + (1.54 * comprehension score) + (1.64 * matrix reasoning score) + (1.48 * similarities score) + (0.98 * picture arrangement score). If the WAIS score was not available (BPD *n* = 12 and NPC *n* = 1) an estimation of the IQ was made based on the education level, using the regression equation from our present sample per group; IQ BPD = 85.932 + (3.360 * ISCED code) and IQ NPC = 85.996 + (3.659 * ISCED code). These regression equations explained respectively 22% and 31% of the variance in IQ. Level of education of both the Dutch and German educational systems were transformed into the International Standard Classification of Education (ISCED).

*Borderline Personality Disorder Severity Index (BPDSI)* - The BPDSI is a semi-structured clinical interview assessing frequency and severity of BPD manifestations ([Arntz et al. 2003](#_ENREF_2); [Giesen-Bloo et al. 2010](#_ENREF_7); [Kroger et al. 2013](#_ENREF_8)). The 70-items reflect the nine BPD criteria described in the DSM-IV. For each item the frequency of the last three months is rated on an 11-point scale, ranging from 0 (never) to 10 (daily). The scores on the subscales provide information on the severity of each of the DSM-IV dimensions, derived by averaging the items scores. The total score is the sum of the nine dimensions scores, ranging from 0 to 90, with an internal consistency of Cronbach’s *α* = 0.93 and subscales ranged between 0.41 and 0.83 ([Arntz et al. 2003](#_ENREF_2)). A total score of 20 distinguishes BPD from other personality disorders ([Giesen-Bloo et al. 2010](#_ENREF_7)).

**Supplementary Results**

**Analyses of BPD patients compared to both control groups of inhibition**

The whole brain random effects ANOVA F-map: inhibition (no-go vs. go) x group (BPD vs. NPC) resulted in three clusters, which were anatomically identified at the left hippocampus (peak Tal: x = -33, y = -19, z = -8; 501 mm^3^; *F*_1,85_ = 9.55, *p* = 0.003), left posterior cingulate cortex (peak Tal: x = -27, y = -70, z = 7; BA 32; 429 mm^3^; *F*_1,85_ = 13.33 *p* < 0.001), and the left superior temporal gyrus (peak Tal: x = 33, y = -49, z = 13; BA 22; 636 mm^3^; *F*_1,85_ = 17.59, *p* < 0.001). Simple effects showed less activity in the NPC for no-go versus go in all three clusters. Furthermore, in the posterior cingulate cortex and superior temporal gyrus BPD patients showed more activity for no-go versus go. These two brain areas also showed a differences for the comparison of BPD and CCP (*F*_1,71_ = 5.64, *p* = 0.020; *F*_1,71_ = 4.26, *p* = 0.043, respectively). Simple effects of the posterior cingulate cortex showed more activity of the BPD compared to the CCP during both no-go and go. There were no confounding effects of medication, age or site.

**Effect of site**

The reported clusters, except for the cluster of the Brainstem, did not show overlap with the significant clusters of group x stimulus x site interaction at lenient significance level of *p* < 0.05 (Fig. S1). Also more detailed analyses within SPSS did not show a significant group x stimulus x site interaction, again with the exception of the brainstem, and the group x stimulus remained significant (Table S1).


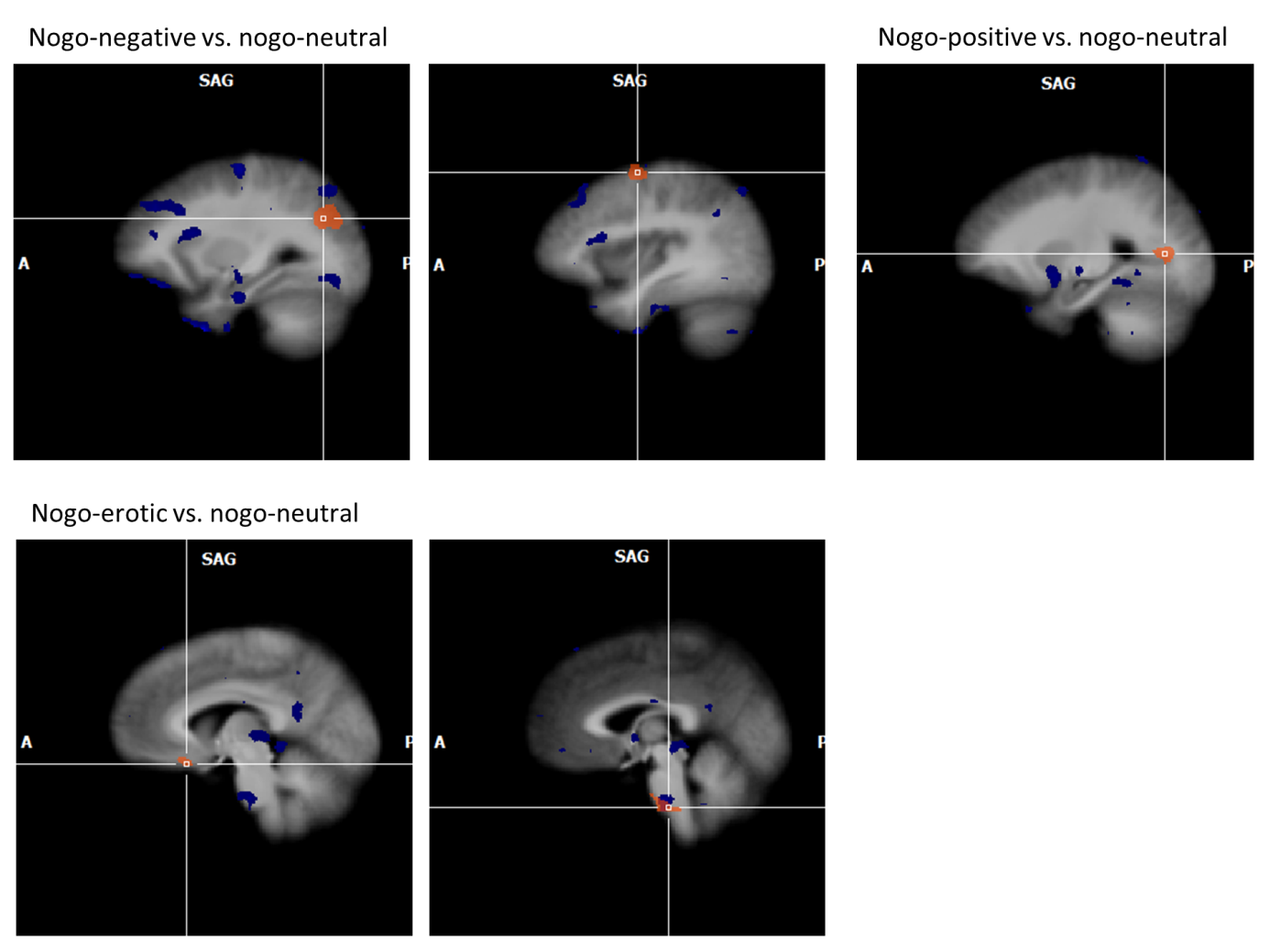
**Fig. S1** Overlap of the reported clusters of group x stimulus x site interaction.

**Table S1.** Detailed analyses of Site.

|  | Stimulus x Group x Site | | Stimulus x Group | |
| --- | --- | --- | --- | --- |
|  | *F* | *p* | *F* | *p* |
| **Nogo Negative versus Nogo Neutral** |  |  |  |  |
| Inferior parietal lobe | 0.81 | 0.673 | 7.60 | 0.006 |
| Middle frontal gyrus, Frontal eye fields | 0.97 | 0.474 | 13.84 | < 0.001 |
| **Nogo Positive versus Nogo Neutral** |  |  |  |  |
| Posterior cingulate cortex | 1.18 | 0.292 | 13.40 | < 0.001 |
| **Nogo Erotic versus Nogo Neutral** |  |  |  |  |
| Subcallosal gyrus, ventromedial | 0.68 | 0.768 | 4.27 | 0.039 |
| Brainstem | 2.98 | < 0.001 | 3.89 | 0.049 |

**References**

Arntz, A., & Dreessen, L. (1995). *BPD-Klachtenlijst 47 [BPD Checklist]*. The Netherlands: Maastricht University.

Arntz, A., van den Hoorn, M., Cornelis, J., Verheul, R., van den Bosch, W. M., & de Bie, A. J. (2003). Reliability and validity of the borderline personality disorder severity index. *Journal of Personality Disorders, 17*(1), 45-59, doi:10.1521/pedi.17.1.45.24053.

Bradley, M. M., & Lang, P. J. (1994). Measuring emotion: the Self-Assessment Manikin and the Semantic Differential. *Journal of Behavior Therapy and Experimental Psychiatry, 25*(1), 49-59, doi:10.1016/0005-7916(94)90063-9.

De Beurs, E. (2004). *Handleiding bij de Brief Symptom Inventory (BSI)*. Leiden: Pits Publishers.

De Beurs, E., & Zitman, F. G. (2006). De Brief Symptom Inventory (BSI): De betrouwbaarheid en validiteit van een handzaam alternatief voor de SCL-90 [The Brief Symptom Inventory (BSI): The reliability and validity of a brief alternative of the SCL-90]. *Maandblad Geestelijke Volksgezondheid, 61*, 120-141.

Derogatis, L. R. (1993). *BSI Brief Symptom Inventory: Administration, Scoring, and Procedure Manual* (4th ed.). Minneapolis, MN: National Computer Systems.

Giesen-Bloo, J. H., Wachters, L. M., Schouten, E., & Arntz, A. (2010). The Borderline Personality Disorder Severity Index-IV: psychometric evaluation and dimensional structure. *Personality and Individual Differences, 49*(2), 136-141, doi:10.1016/j.paid.2010.03.023.

Kroger, C., Vonau, M., Kliem, S., Roepke, S., Kosfelder, J., & Arntz, A. (2013). Psychometric properties of the German version of the borderline personality disorder severity index--version IV. *Psychopathology, 46*(6), 396-403, doi:10.1159/000345404.

Lobbestael, J., Arntz, A., Harkema-Schouten, P., & Bernstein, D. (2009). Development and psychometric evaluation of a new assessment method for childhood maltreatment experiences: the interview for traumatic events in childhood (ITEC). *Child Abuse & Neglect, 33*(8), 505-517, doi:10.1016/j.chiabu.2009.03.002.

Schrimsher, G. W., O'Bryant, S. E., O'Jile, J. R., & Sutker, P. B. (2008). Comparison of tetradic WAIS-III short forms in predicting full scale IQ scores in neuropsychiatric clinic settings. *Journal of Psychopathology and Behavioral Assessment, 30*(3), 235-240, doi:10.1007/s10862-007-9066-9.

Stiglmayr, C. E., Shapiro, D. A., Stieglitz, R. D., Limberger, M. F., & Bohus, M. (2001). Experience of aversive tension and dissociation in female patients with borderline personality disorder -- a controlled study. *Journal of Psychiatric Research, 35*(2), 111-118, doi:10.1016/S0022-3956(01)00012-7.

van Zutphen, L., Siep, N., Jacob, G. A., Domes, G., Sprenger, A., Willenborg, B., et al. (2017). Always on guard: emotion regulation in women with borderline personality disorder compared to nonpatient controls and patients with cluster-C personality disorder. *Journal of Psychiatry & Neuroscience, 43*(1), 37–47, doi:10.1503/jpn.170008.
